# Effects of age and knee osteoarthritis on the modular control of walking: A pilot study

**DOI:** 10.1371/journal.pone.0261862

**Published:** 2021-12-29

**Authors:** Sarah A. Roelker, Rebekah R. Koehn, Elena J. Caruthers, Laura C. Schmitt, Ajit M. W. Chaudhari, Robert A. Siston

**Affiliations:** 1 Department of Kinesiology, University of Massachusetts Amherst, Amherst, Massachusetts, United States of America; 2 Department of Mechanical and Aerospace Engineering, The Ohio State University, Columbus, Ohio, United States of America; 3 Department of Engineering, Otterbein University, Westerville, Ohio, United States of America; 4 School of Health and Rehabilitation Sciences, The Ohio State University, Columbus, Ohio, United States of America; 5 Sports Medicine Research Institute, The Ohio State University Wexner Medical Center, Columbus, Ohio, United States of America; 6 Division of Physical Therapy, School of Health and Rehabilitation Sciences, The Ohio State University, Columbus, Ohio, United States of America; 7 Department of Biomedical Engineering, The Ohio State University, Columbus, Ohio, United States of America; 8 Department of Orthopaedics, The Ohio State University, Columbus, Ohio, United States of America; University of Illinois at Urbana-Champaign, UNITED STATES

## Abstract

**Background:**

Older adults and individuals with knee osteoarthritis (KOA) often exhibit reduced locomotor function and altered muscle activity. Identifying age- and KOA-related changes to the modular control of gait may provide insight into the neurological mechanisms underlying reduced walking performance in these populations. The purpose of this pilot study was to determine if the modular control of walking differs between younger and older adults without KOA and adults with end-stage KOA.

**Methods:**

Kinematic, kinetic, and electromyography data were collected from ten younger (23.5 ± 3.1 years) and ten older (63.5 ± 3.4 years) adults without KOA and ten adults with KOA (64.0 ± 4.0 years) walking at their self-selected speed. Separate non-negative matrix factorizations of 500 bootstrapped samples determined the number of modules required to reconstruct each participant’s electromyography. One-way Analysis of Variance tests assessed the effect of group on walking speed and the number of modules. Kendall rank correlations (*τ*_*b*_) assessed the association between the number of modules and self-selected walking speed.

**Results:**

The number of modules required in the younger adults (3.2 ± 0.4) was greater than in the individuals with KOA (2.3 ± 0.7; *p* = 0.002), though neither cohorts’ required number of modules differed significantly from the unimpaired older adults (2.7 ± 0.5; *p* ≥ 0.113). A significant association between module number and walking speed was observed (*τ*_*b*_ = 0.350, *p* = 0.021) and individuals with KOA walked significantly slower (0.095 ± 0.21 m/s) than younger adults (1.24 ± 0.15 m/s; *p* = 0.005). Individuals with KOA also exhibited altered module activation patterns and composition (which muscles are associated with each module) compared to unimpaired adults.

**Conclusion:**

These findings suggest aging alone may not significantly alter modular control; however, the combined effects of knee osteoarthritis and aging may together impair the modular control of gait.

## Introduction

Functional limitations, including difficulty walking, climbing stairs, or crouching, in community dwelling older adults have been shown to be predictive of future disability [1] as well as falls, pain, and medical expenses [2]. Impaired gait limits function in 32% of older adults and prevalence of gait impairments increases with age [3]. Impaired neuromuscular activity has been identified as a cause of the age-related changes in muscle function that leading to these functional limitations [4–6]. Furthermore, age-related differences in muscle activation patterns have been linked to altered gait kinematics and kinetics in older adults with no history of musculoskeletal injury or joint disorders [7–10]. Older adults exhibit highly repeatable electromyography (EMG) signals, which suggests an inability to adapt their motor control to perturbations and reflects a loss of neural plasticity [7, 10]. This age-related decrease in control complexity and adaptability of the nervous system may indicate that changes to the organization of the neural mechanisms underlying motor control are responsible for impaired functional performance in older adults.

Furthermore, it is important to differentiate between neuromuscular changes associated with normal aging and those associated with age-related disorders. For example, symptomatic knee osteoarthritis (KOA) is a leading cause of disability in older adults [11]; yet, not all older adults develop KOA [12]. Individuals with KOA have difficulty completing activities of daily living [13] and walk with altered kinematics and kinetics compared to unimpaired age-matched individuals. Specifically, individuals with KOA walk with a reduced peak knee flexion angle and reduced peak knee flexion and extension moments [14]. These KOA-related changes in joint mechanics have been suggested to be related to altered muscle activation patterns in the presence of KOA [15], and may be suggestive of a disease-related loss of motor control complexity. For example, individuals with KOA exhibit increased co-contraction of the vastus medialis and medial gastrocnemius compared to unimpaired controls [16]. In addition, subtle differences in the magnitude and shape of the vasti, hamstring, and gastrocnemius activation patterns are capable of distinguishing between asymptomatic adults and individuals with mild to moderate (Kellgren-Lawrence (KL) grades I-III [17]) and severe (KL IV) KOA [18–20], which may indicate changes in motor control strategy with increasing disease severity.

Current motor control theory suggests the underlying mechanisms controlling muscle activity during gait can be decomposed into a few “primitive signals” [21–23] that represent basic central features of the motor programs [21]. Muscles are grouped into modules (sometimes referred to as synergies) based on the similarity of their activation patterns to the temporal pattern of the primitive signals. A greater number of modules required to represent the original muscle activation patterns suggests a more complex neuromuscular control strategy [24]. Weighting factors are assigned to the modules to quantify the strength of each muscle’s representation within a given module. Modular organization (muscle weightings and temporal patterns) has been shown to be consistent across walking speeds in healthy adults [23, 24], which indicates these modules are a robust representation of the underlying neuromuscular control of gait [25].

Although modular control of gait has been characterized in healthy younger adults (age range: 26 to 42 years) [23, 26] and healthy older adults (63.1 ± 9.1 years) [24, 25, 27], differences in the factorization method and the number of muscles included in the factorization used to identify modules in younger and older adults may limit accurate comparison of the modular control of gait between age-groups. Only one study by Monaco et al. [28] has directly compared the modular control of gait between age-groups. Although the authors found no significant differences in module muscle weightings and temporal patterns between younger and older adults during gait, the authors prescribed the number of modules extracted to be equal to 5 for all participants prior to performing their factor analysis, which may have hindered their ability to identify differences in modular control between age-groups. Alternatively, investigations of age-related differences in muscle synergies controlling postural responses during step preparation demonstrated diminished control of gait initiation in older adults compared to younger adults [29, 30], which suggests older adults utilize altered modular control strategies.

One recent study investigated the modular control of patients who had undergone a total knee arthroplasty (TKA), the end-stage treatment for KOA [31]. Greater modular control complexity was observed in high-functioning compared to low-functioning TKA patients, although both TKA groups exhibited reduced complexity compared to unimpaired controls [31]. However, whether modular control was altered due to the disease prior to surgery or was altered as a result of surgery is unknown. It is possible that the observed differences between KOA and asymptomatic individuals’ muscle activation patterns [18–20] are due to changes in the underlying motor control strategy.

Together, the altered muscle activity observed in older adults compared to younger adults and in individuals with KOA compared to unimpaired older adults suggests that modular control may differ between age groups as well as between healthy and impaired older adults. The purpose of this pilot study was to determine if the modular control of gait differs between healthy younger and older adults and between healthy older adults and individuals with end-stage KOA. We hypothesized that there would be no differences in locomotor complexity, quantified by the number of modules required to reconstruct the experimental activation patterns, between younger and older adults without KOA, but that the modular organization of the older adults would be different than the younger adults. In addition, we hypothesized that the muscle activation patterns of individuals with end-stage KOA would reduce to fewer modules than that of the younger and older adults without KOA, representing a reduction in neuromuscular control complexity. By differentiating between age- and pathology-related changes to the modular control of gait, this study may provide insight into the neurological mechanisms underlying reduced locomotor performance in older adults and individuals with KOA.

## Materials and methods

### Data collection

In this cross-sectional study, data from ten younger adults (5 female; 23.5 ± 3.1 years; 1.8 ± 0.1 m; 71.2 ± 10.0 kg), ten unimpaired older adults (5 female; 63.5 ± 3.4 years; 1.7 ± 0.1 m; 71.5 ± 13.5 kg), and ten individuals with primarily medial compartment KOA (7 female; 64.0 ± 4.0 years; 1.7 ± 0.1 m; 89.7 ± 7.5 kg) were included as a secondary analysis of previously collected data. Participants in the younger and unimpaired older adult cohorts were recruited from the local Columbus, OH area through fliers, email, and word of mouth. The unimpaired younger and older adults were included if they were able to walk without pain or a limp, had no history of serious injury to either leg that required surgery or involved a ligament, tendon, muscle or meniscus tear. In addition, younger adults had to be at least 18 years of age at the time of testing and not in their second or third trimester of pregnancy. The older adults were required to be between 55 and 75 years of age. Young adults were recruited in 2013 and data collection took place from April 2013 to March 2014. Unimpaired older adults were recruited in 2016 and data collection took place from August to October 2016. For the cohort of individuals with KOA, three orthopaedic surgeons identified potential participants based on consultation for a TKA at The Ohio State Wexner Medical Center. The individuals with KOA included in this study are a subset of participants from a larger study investigating gait biomechanics before and after TKA [32, 33]. Individuals with predominantly medial compartment tibiofemoral osteoarthritis were included if they were scheduled for primary TKA within 8 weeks of the data collection. Individuals with KOA were also required to have a body mass index (BMI) less than 45, the ability to walk 20 m without an assistive device, and no history of previous TKA or osteotomy. Recruitment for the individuals with KOA began in April 2012 on a rolling basis and data was collected from June 2012 to February 2015.

All participants provided written informed consent prior to participating in the data collection. To protect participant privacy, anonymity was assured through coding of all collected data. All study procedures were approved by The Ohio State University Institutional Review Board and adhered to the ethical principles for medical research involving human subjects outlined in the Declaration of Helsinki. All data collection was performed in the Clinical, Functional, and Performance Biomechanics Lab at The Ohio State University.

All participants performed at least five over-ground walking trials at their self-selected speeds. Self-selected speed was calculated as the distance between heel-markers during consecutive ipsilateral heel-strikes divided by the time between heel-strikes. Skin-mounted reflective markers were applied to the upper and lower extremities according to the modified Full-Body Point-Cluster Technique (PCT) to measure full body kinematics [34, 35]. The three-dimensional trajectories of the reflective markers were collected at 150 Hz using ten Vicon MX-F40 cameras (Vicon, Los Angeles, CA). An embedded-force-plate walkway formed by 6 force platforms (Bertec, Columbus, OH) collected ground reaction forces (GRFs) at 1500 Hz. Surface electromyography (EMG) (Telemyo DTS System, Noraxon, Scottsdale, AZ) was collected at 1500 Hz from 8 muscles on each participant’s dominant (younger and older adults) or involved (KOA) limb to measure muscle activation patterns from the rectus femoris, vastus lateralis, vastus medialis, biceps femoris, medial hamstrings, lateral gastrocnemius, medial gastrocnemius, and soleus. The skin over each muscle was prepared by shaving hair from the skin and cleansing the area with alcohol. Ag/AgCl dual-electrodes (Vermed, Buffalo, NY, Peripheral Nerve Stimulation Dual Element Electrodes, rectangular, 1.625 in x 3.25 in, 0.42 in sensor diameter, 1.625 in inter-electrode distance) were placed over the belly of each muscle. To determine the dominant limb of the healthy younger and older adults, each participant was asked which leg he or she would use to kick a ball [36].

### KOA-specific data collection procedures

For each individual with KOA, two fellowship-trained, musculoskeletal radiologists (JP and AR; see acknowledgements) graded the patient’s tibiofemoral radiographic severity using the KL grading system [17] by consensus. In addition to the walking trials, the individuals with KOA performed three clinical performance-based assessments: the six-minute walk test (6MW) [37], the timed stair climbing test (SCT) [38], and the timed-up and go test (TUG) [39]. Finally, self-reported function was evaluated using four subscales of the Knee Injury and Osteoarthritis Outcome Score (KOOS) [40]: pain, symptoms, activities of daily living (ADL), and knee-related quality of life (QOL). Each subscale is evaluated independently with scores between 0 and 100, with higher scores indicating better self-perceived function.

### Identification of muscle modules

Nonnegative Matrix Factorization (NMF) [41] was chosen to identify modules because it produces additive components that can be interpreted physiologically [42] given that neuron firing rates are never negative and synaptic strengths do not change sign [41]. For each participant, EMG from a minimum of 5 gait cycles (mean: 11.3 ± 4.8 cycles; range: 5–23 cycles) were included in the analysis. The EMG data were demeaned, passed through a 30–300 Hz sixth order Butterworth band-pass filter, and full wave rectified. The data were then passed through a sixth order Butterworth low-pass filter at 6 Hz to create linear envelopes. Each muscle’s processed EMG data were normalized to the maximum EMG activation value from all walking trials. For each gait cycle, the normalized EMG was time normalized to 201 time points, accounting for every 0.5% of the gait cycle. Then, the normalized EMG from all gait cycles, *g*, were concatenated into an *m x t* matrix (EMG_o_), where *m* indicates the number of muscles (8) and *t* indicates the total number of time points (201 x *g*). Each muscle’s concatenated EMG in EMG_o_ was normalized to unit variance. An NMF algorithm defined by Lee and Seung (1999) was applied to the *m x t* matrix. NMF defines the modular organization by populating an *m x n* matrix representing the relative weighting of each muscle within each module, *n*, (Weighting Matrix) and an *n x t* matrix reflecting the temporal activation of the module across the gait cycle (Pattern Matrix). NMF assumes that muscles may belong to more than one module and that the muscle weightings on each module are fixed throughout the gait cycle. The two matrices were multiplied to produce an *m x t* matrix of the reconstructed activation patterns (EMG_r_). A multiplicative update algorithm using 50 replicates and a maximum of 10000 iterations adjusted the muscle weightings and activation profiles to minimize the sum of squared errors between EMG_o_ and EMG_r_.

For each participant in each population, separate NMF analyses extracted *n* modules from EMG_o_, where *n* ranged from 1 to 8 modules. For each extraction, the percent variability accounted for (VAF) by EMG_r_ was calculated as a measure of the agreement between the original and reconstructed activation patterns. The VAF for each muscle, *m*, (mVAF) was calculated as:

$$mVAF\;(\%)=\left(1-\frac{{\left({EMG}_{o}^{m}-{EMG}_{r}^{m}\right)}^{2}}{{\left({EMG}_{o}^{m}\right)}^{2}}\right)\times 100,$$ (1)

where ${EMG}_{o}^{m}$ is the original EMG of muscle, *m*, and ${EMG}_{r}^{m}$ is the reconstructed EMG of muscle, *m*. The total VAF (tVAF) was calculated as:

$$tVAF\;(\%)=\left(1-\frac{\sum_{m=1}^{8}{\left({EMG}_{o}^{m}-{EMG}_{r}^{m}\right)}^{2}}{\sum_{m=1}^{8}{\left({EMG}_{o}^{m}\right)}^{2}}\right)\times 100,$$ (2)

where for each of the eight muscles, *m*, ${EMG}_{o}^{m}$ is the original EMG of muscle, *m*, and ${EMG}_{r}^{m}$ is the reconstructed EMG of muscle, *m*.

The 95% confidence interval (CI) of the VAF was determined using a bootstrapping procedure [43]. For each number of modules extracted (1 to 8), the EMG_o_ matrix was resampled 500 times with replacement. For each sample, the muscle mVAFs and the tVAF were calculated and then the 95% CIs of the mVAFs and tVAF were constructed from the bootstrapped VAF values. The number of modules required to adequately reconstruct EMG_o_ was chosen as the minimum number of modules extracted for which the lower bound on the 95% CI for the tVAF was greater than or equal to 90% and the minimum mVAF was greater than 75% or adding another module did not increase the minimum mVAF by more than 5%.

### Module analsyis

Module composition (which muscles are primarily associated with each module) was characterized by the Weighting Matrices from the bootstrapped samples. Since NMF outputs the modules in a random order for each bootstrap sample, the *n* columns of each Weighting Matrix associated with each *n* module must be sorted to ensure consistent module characterization across samples (e.g., the first column/module is always associated with high quadriceps activity, the second column/module is always associated with high plantarflexor activity, etc.). To sort the modules, *k*-means clustering was used to classify the *n* column of each sample’s Weighting Matrix associated with each module using the Weighting Matrix extracted by the NMF from EMG_o_ as the seed. On a sample-by-sample basis, each module’s muscle weightings were normalized to the maximum muscle weighting in the module, which resulted in a muscle weight of 1 for the muscle which had the greatest weighting in the module. Similarly, each participant’s principal patterns were normalized to the peak value so that the magnitude of each pattern ranged from 0 to 1. Then, for each module, the muscle weightings were averaged over the 500 bootstrapped samples so that 95% CIs could be identified for the muscle weightings.

Within each population, common modules between participants were identified using Pearson’s correlation coefficients. Modules from two participants were considered common if the module weightings were correlated with *r* ≥ 0.834, which corresponds to the critical *r*^*2*^ for 8 muscles at *p* = 0.01 [44]. For each module that was common between participants in a population, the module muscle weightings were averaged across all participants with that common module. Between population correlations of average muscle weightings for common within population modules were determined to quantify similarity in module composition across populations. Module composition was also compared between populations based on the number of significantly active muscles per module (W_musc_) and the sum of the contributions of the significantly active muscles in a module (W_sum_; i.e., the sum of the bar heights of the muscle weightings) [45]. Both W_musc_ and W_sum_ quantify the muscle co-activity of the module. A muscle was considered significantly active if the 95% CI for the muscle weighting did not include zero. The activation timing profiles of common modules were compared using the area under the activation curve (AUC). For each participant, the average activation timing profile from the *g* gait cycles included in the analysis was determined for each common module. Then, the AUC was calculated for six gait phases: weight acceptance (0–15% gait cycle), early midstance (15–30% gait cycle), late midstance (30–50% gait cycle), terminal stance (50–65% gait cycle), early swing (65–80% gait cycle), and late swing (80–100% gait cycle).

### Joint kinematic and kinetic analysis

Sagittal plane joint angle and internal joint moment profiles for the hip, knee, and ankle were determined for each participant from a representative gait cycle and then averaged within each population. Prior to averaging, each individual’s joint moments were normalized by dividing the joint moment by body weight, height, and self-selected walking speed. At the hip, peak angles were calculated for early stance hip flexion (H1), late stance hip extension (H2), and swing phase hip flexion (H3) and peak moments were calculated for early stance hip extension (HM1), late stance hip flexion (HM2), and swing phase hip extension (HM3). At the knee, peak angles were calculated for early stance knee flexion (K1), midstance knee extension (K2), and swing phase knee flexion (K3) and peak moments were calculated for weight acceptance knee flexion (KM1), early stance knee extension (KM2), midstance knee flexion (KM3), late stance knee extension (KM4), and swing phase knee flexion (KM5). At the ankle, peak angles were calculated for early stance plantarflexion (A1), late stance dorsiflexion (A2), push-off plantarflexion (A3), and swing phase dorsiflexion (A4); and peak moments were calculated for early stance dorsiflexion (AM1) and late stance plantarflexion (AM2). Peak measures were averaged across participants within each population.

### Statistics

A significance level of α<0.05 was set *a priori* for all statistical tests. All statistics analyses were performed in MATLAB 2016a (MathWorks, Natick, MA). The Lilliefors test was used to assess the normality of the data and the Bartlett test was used to test for homogeneity of variances between groups. Individual one-way Analysis of Variance (ANOVA) tests were used to assess the effect of group on height, mass, BMI, self-selected walking speed, stride length normalized to leg length, W_sum_ and AUC for common modules, and peak joint angles and moments. The assumption of normality was not satisfied for age, the number of modules required to adequately reconstruct the EMG, or W_musc_; therefore, the Kruskal-Wallis Test was used to assess differences in these variables between groups. When appropriate, Bonferroni post-hoc analyses were used to test for pairwise differences. The effect of the absence of a common module typically found in a healthy young adult (due to merging with another module) on peak joint kinematics and kinetics was assessed by two-sample t-tests between those participants with and without an independent common module, regardless of population. Participants who did not have a common module because the control of the primary muscles in the module was separated into two modules were excluded from this particular analysis. The group mean and standard deviation for demographic variables, number of modules, W_musc_, W_sum_, AUC, and peak joint kinematic and kinetics are reported.

For the set of all participants and each population individually, Kendall rank correlations (Kendall’s *τ*_*b*_) assessed the association between the number of required modules and self-selected walking speed. For the KOA group, Kendall rank correlations were also used to assess the association between the number of required modules and their clinical performance-based test and KOOS scores. Also, for the KOA group, Spearman’s rank correlation was used to determine whether the number of required modules and KL grade were correlated. Correlation coefficients are reported for these analyses.

## Results

### Population average demographics and activation patterns

Compared to the younger and older adults, the individuals with KOA had significantly greater mass (all pairwise *p ≤* 0.002) and BMI (all pairwise *p* < 0.001) (Table 1). On average, the KOA group also walked significantly slower (pairwise *p* = 0.005) and with a shorter stride length (pairwise *p* = 0.010) than the younger adults. There was no statistically significant difference in age between the older adult and KOA groups (pairwise *p* = 0.946). As expected, the older adults and individuals with KOA were significantly older than the younger adults (all pairwise *p* < 0.001).

**Table 1:** Population demographics.

	Younger (n = 10)	Older (n = 10)	KOA (n = 10)	p-Value	Test Statistic
Sex	5M, 5F	5M, 5F	3M, 7F		
Age (years)*	23.5 ± 3.1	63.5 ± 3.4	64.0 ± 4.0	< 0.001^a^^,^^b^	Χ^2^_(2, N = 30)_ = 19.51
Height (m)	1.75 ± 0.07	1.68 ± 0.08	1.70 ± 0.10	0.209	F_2,27_ = 1.66
Mass (kg)	71.2 ± 10.0	71.5 ± 13.5	89.7 ± 7.5	< 0.001^b^^,^^c^	F_2,27_ = 9.87
BMI (kg/m^2^)	23.1 ± 2.8	24.8 ± 2.6	32.3 ± 4.1.9	< 0.001^b^^,^^c^	F_2,27_ = 23.11
Speed (m/s)	1.24 ± 0.15	1.13 ± 0.20	0.95 ± 0.21	0.007^b^	F_2,27_ = 6.06
Stride Length (Normalized by Leg Length)	1.68 ± 0.17	1.51 ± 0.17	1.37 ± 0.28	0.013^b^	F_2,27_ = 5.08
Number of Modules*	3.2 ± 0.4	2.7 ± 0.5	2.3 ± 0.7	0.006^b^	Χ^2^_(2, N = 30)_ = 10.14

* Kruskal-Wallis Test was used to assess differences in these variables between groups. ANOVAs were used to test for differences between groups for all other variables. Symbols indicate statistically significant pairwise differences determined by Bonferroni tests between

^a^ Younger and Older.

^b^ Younger and KOA.

^c^ Older and KOA.

**Fig 1:**
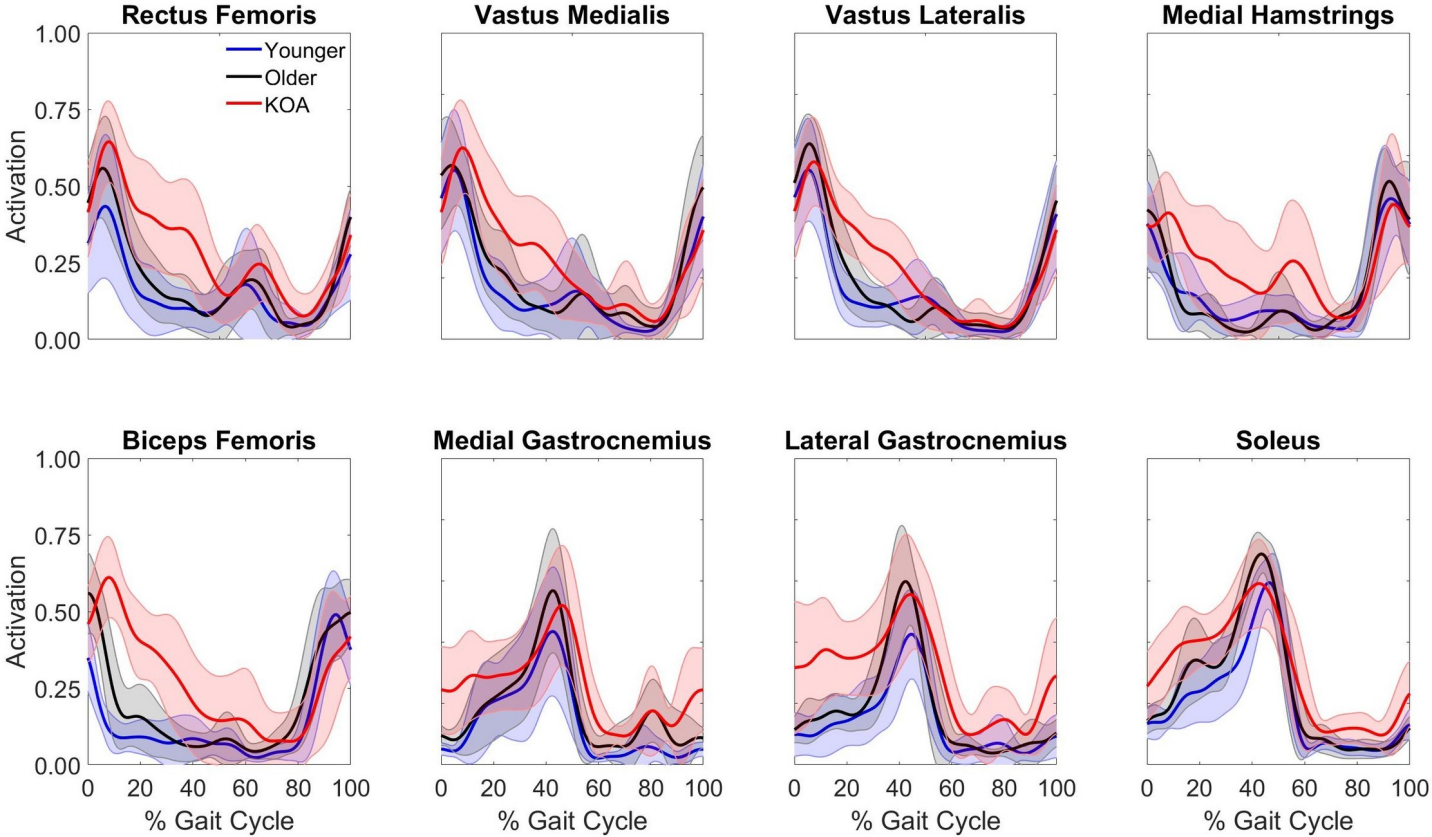
Average normalized EMG of young adults, older adults, and KOA patients. Solid lines represent population average. Shaded areas represent ± one standard deviation.

The normalized EMG of the eight muscles exhibited similar activation patterns across populations (Fig 1). However, the normalized activation magnitude of the KOA group tended to be higher during stance compared to that of the older and younger adults across all muscles. Swing phase activation magnitudes were similar across all three groups.

### Modular control complexity

Fewer modules were required to reconstruct the EMG of the KOA group (2.3 ± 0.7 modules) than the younger adult group (3.2 ± 0.4 modules) (pairwise *p* = 0.005; Table 1). The number of modules required for the older adults (2.7 ± 0.5 modules) did not significantly differ from the other two groups (all pairwise *p* ≥ 0.201). Of the 10 younger adults, eight participants required three modules and two required four modules. Of the 10 older adults, three participants required two modules and seven required three modules. Of the 10 individuals with KOA, one participant required one module, five required two modules, and four required three modules.

**Fig 2:**
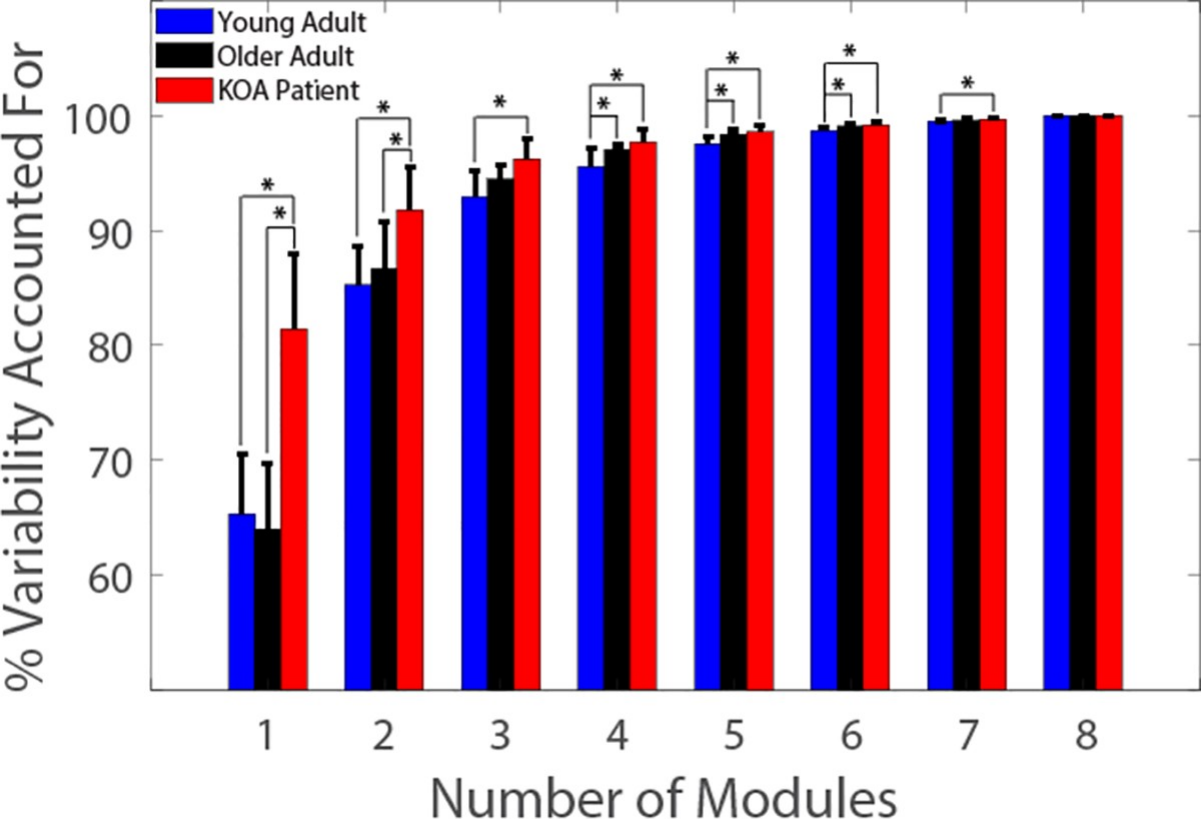
Total variability accounted for by *n* modules. * indicate significant pairwise differences between populations.

There were significant differences in minimum VAF between groups when one through seven modules were extracted by NMF (Fig 2; 1 Module: F_2,27_ = 27.81, *p* < 0.001; 2 Modules: F_2,27_ = 8.42, *p* = 0.001; 3 Modules: F_2,27_ = 8.27, *p* = 0.002; 4 Modules: F_2,27_ = 8.58, *p* = 0.001; 5 Modules: F_2,27_ = 9.93, *p* < 0.001; 6 Modules: F_2,27_ = 8.01, *p* = 0.002; 7 Modules: F_2,27_ = 6.56, *p* = 0.005). The KOA group had significantly greater VAF than the younger and older adults when one module (both pairwise *p* < 0.001) and two modules (both pairwise *p* ≤ 0.014) were extracted. The KOA group also had significantly greater VAF than the younger adults when three (pairwise *p* = 0.001), four (pairwise *p* = 0.001), five (pairwise *p* < 0.001), six (pairwise *p* = 0.002), and seven (pairwise *p* = 0.005) modules were extracted. The older adults had significantly greater VAF than the younger adults when four (pairwise *p* = 0.029), five (pairwise *p* = 0.007), and six (pairwise *p* = 0.025) modules were extracted.

### Module composition and activation timing

**Fig 3:**
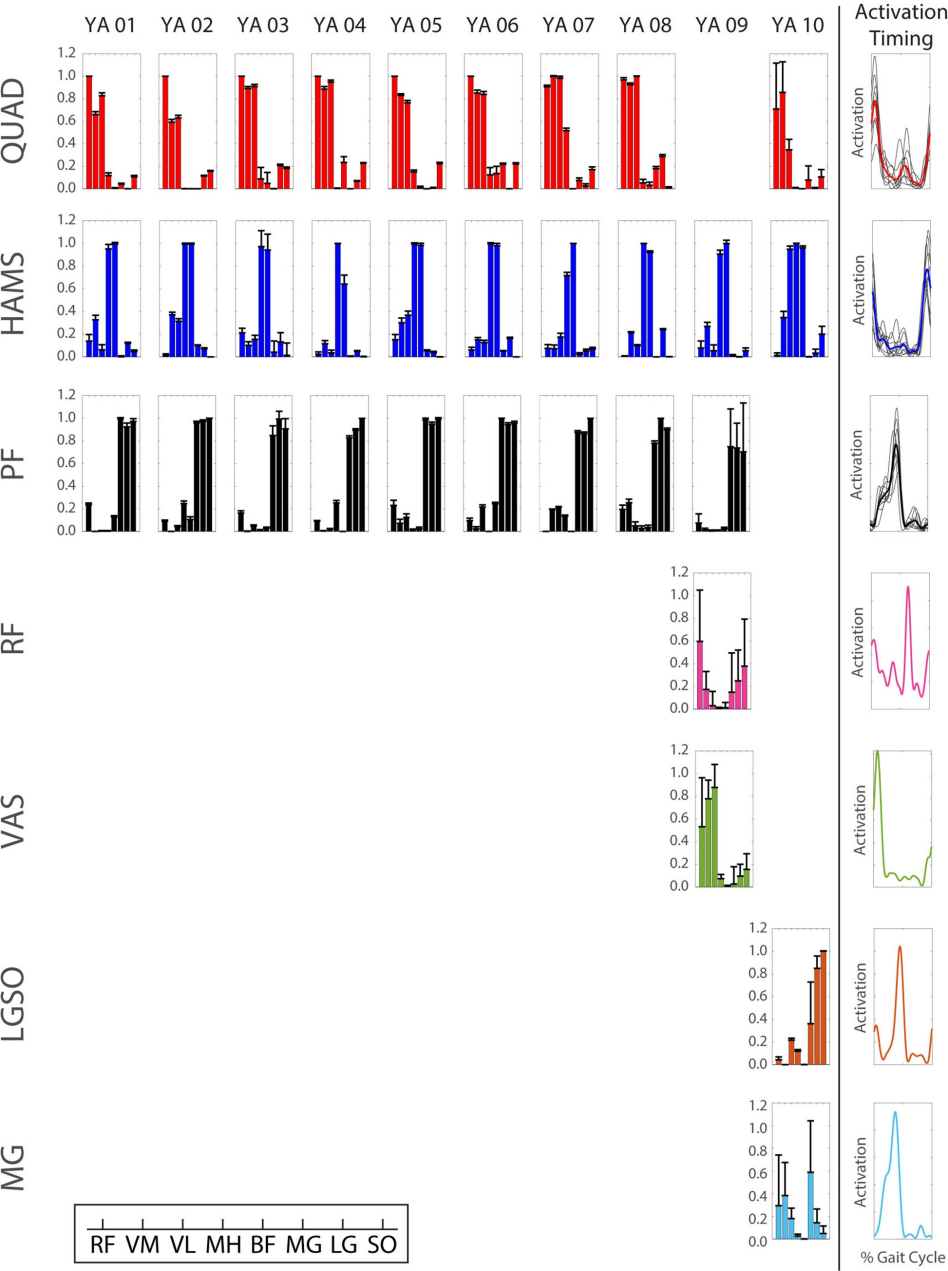
Young adult (YA) modular control. Bar heights represent the average bootstrapped muscle weights for the respective subject for the respective module. Error bars represent standard deviation of the bootstrapped weights. Bold, colored curves represent average activation timing profile across all subjects with the respective module. Gray curves represent individual subject activation timing profiles. Muscle abbreviations: rectus femoris (RF), vastus medialis (VM), vastus lateralis (VL), medial hamstrings (MH), biceps femoris (BF), medial gastrocnemius (MG), lateral gastrocnemius (LG), soleus (SO).

**Fig 4:**
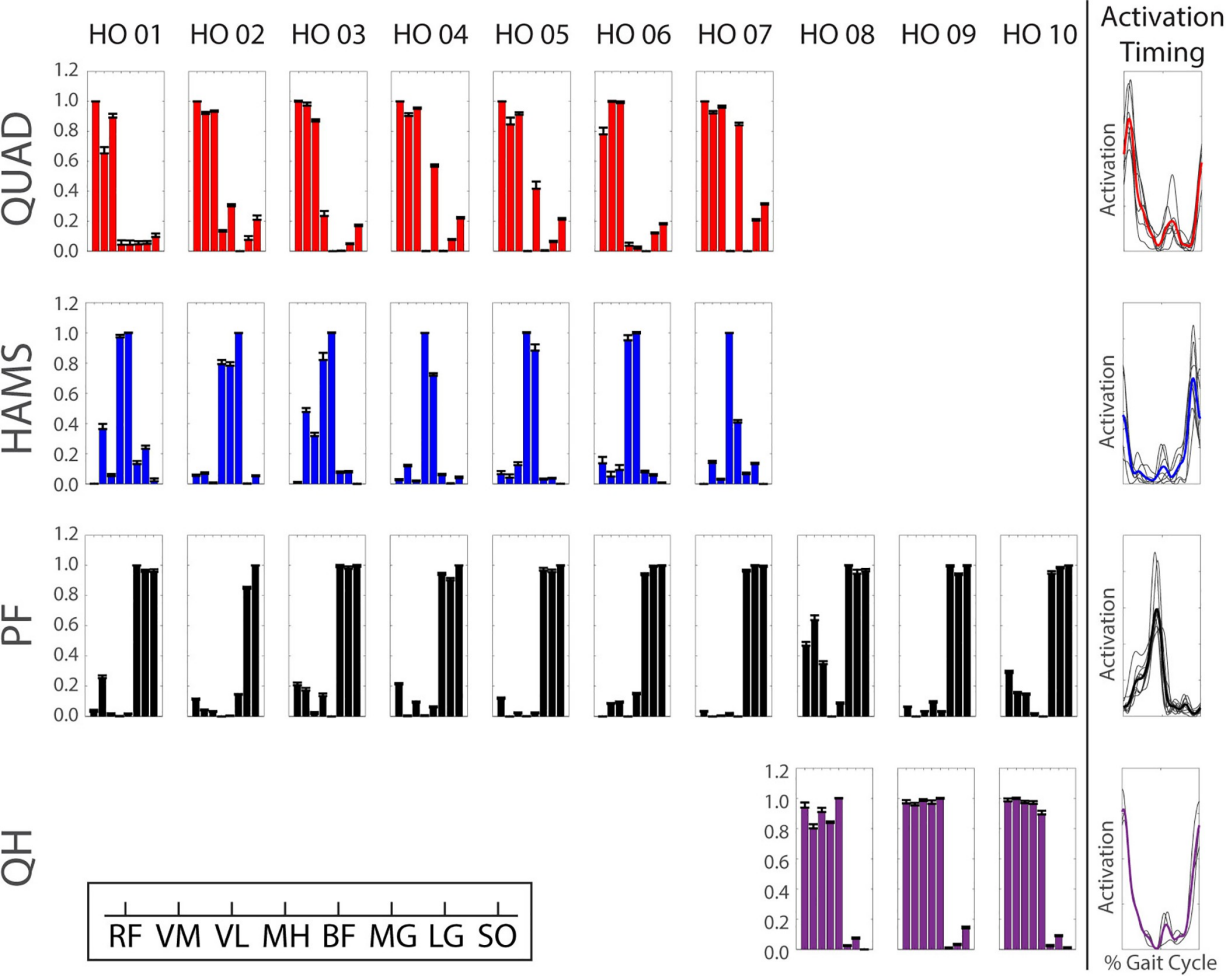
Healthy older (HO) adult modular control. Bar heights represent the average bootstrapped muscle weights for the respective subject for the respective module. Error bars represent standard deviation of the bootstrapped weights. Bold, colored curves represent average activation timing profile across all subjects with the respective module. Gray curves represent individual subject activation timing profiles. Muscle abbreviations: rectus femoris (RF), vastus medialis (VM), vastus lateralis (VL), medial hamstrings (MH), biceps femoris (BF), medial gastrocnemius (MG), lateral gastrocnemius (LG), soleus (SO).

**Fig 5:**
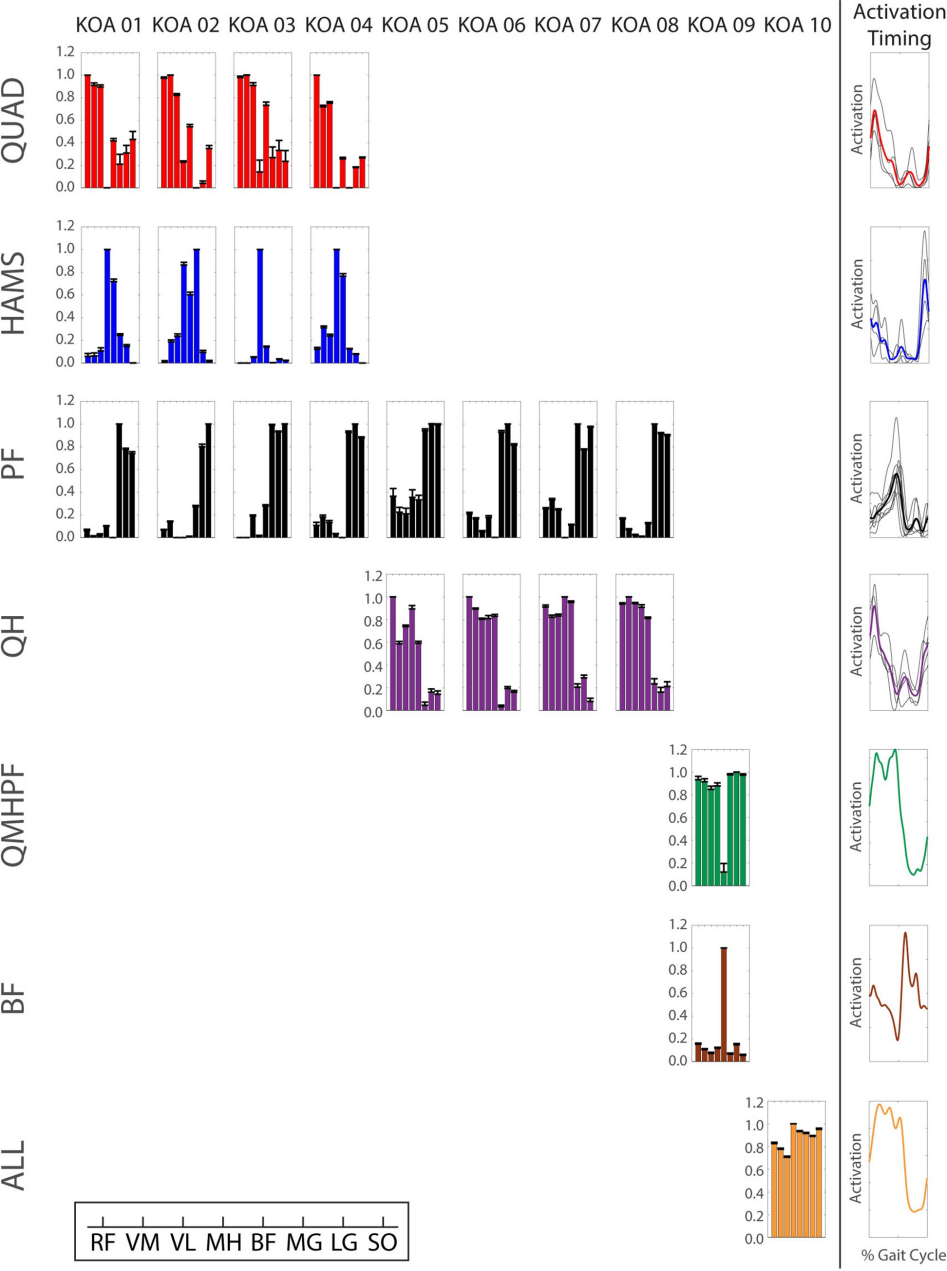
KOA patient modular control. Bar heights represent the average bootstrapped muscle weights for the respective subject for the respective module. Error bars represent standard deviation of the bootstrapped weights. Bold, colored curves represent average activation timing profile across all subjects with the respective module. Gray curves represent individual subject activation timing profiles. Muscle abbreviations: rectus femoris (RF), vastus medialis (VM), vastus lateralis (VL), medial hamstrings (MH), biceps femoris (BF), medial gastrocnemius (MG), lateral gastrocnemius (LG), soleus (SO).

Although the total number of modules differed between subjects across the populations, four common modules were identified between at least two participants within two or more populations (Table 2; Figs 3–5): 1) the quadriceps (QUAD) module characterized by high muscle weightings for the rectus femoris, vastus medialis, and vastus lateralis, 2) the hamstrings (HAMS) module characterized by high muscle weightings for the medial hamstrings and biceps femoris, 3) the plantarflexor (PF) module characterized by high muscle weightings for the medial and lateral gastrocnemius and soleus, and 4) the merged quadriceps and hamstrings (QH) module characterized by high muscle weightings for the rectus femoris, vastus medialis, vastus lateralis, medial hamstrings, and biceps femoris. Of the 30 total participants in the study, 20 participants exhibited the QUAD module (9 younger, 7 older, and 4 KOA participants), 19 participants exhibited the HAMS module (10 younger, 6 older, and 3 KOA participants), 26 participants exhibited the PF module (9 younger, 9 older, and 8 KOA participants), and 7 participants exhibited the QH module (0 younger, 3 older, and 4 KOA participants). These participants were included in the correlation of common modules between populations and the comparisons of W_sum_, W_musc_, and AUC between populations.

**Table 2:** Common module pearson correlation coefficients between populations.

Module	QUAD		HAMS		PF		QH
Population	Older	KOA	Older	KOA	Older	KOA	KOA
Younger	0.970	0.931	0.990	0.895	0.996	0.998	
Older		0.982		0.932		0.999	0.990

There were strong correlations in the muscle weightings between all three populations for the QUAD (*r* ≥ 0.931), HAMS (*r* ≥ 0.895), and PF (*r* ≥ 0.996) modules (Table 2). The QH module was only observed in older adults (n = 3) and KOA participants (n = 4) and there was a strong correlation in the QH muscle weightings between these populations (*r* = 0.990).

Within the group, common modules were generally correlated across all participants who were classified as having the module (Table 3). In the young adults, the common modules were correlated between all participants with that common module. In the older adults, the QUAD modules of all seven individuals with a QUAD module were correlated and the QH modules of all three individuals with a QH module were correlated. However, the HAMS and PF modules of one older adult (healthy older (HO) adult 02) were not correlated with those of the other six and nine older adults who had a HAMS and PF module, respectively. In addition, the HAMS module of one individual with KOA (KOA 02) did not correlate with the HAMS module of the other three individuals with KOA who had a HAMS module. Otherwise, the common modules of the individuals with KOA were correlated between all individuals with that common module.

**Table 3:** Number of participants with correlated module within population*.

Population	Number of Modules**	QUAD Module	HAMS Module	PF Module	QH Module
Younger	3 (n = 8)	8/8	8/8	8/8	0/0
	4 (n = 2)	1/1	2/2	1/1	0/0
Older	2 (n = 3)	0/0	0/0	3/3	3/3
	3 (n = 7)	7/7	6/7	6/7	0/0
KOA	1 (n = 1)	0/0	0/0	0/0	0/0
	2 (n = 5)	0/0	0/0	4/4	4/4
	3 (n = 4)	4/4	3/4	4/4	0/0

* Number of participants within a population with correlated modules / total number of participants within the population classified by K-means clustering to have that module.

** n = number of participants within the population that have X number of modules.

There were no differences in W_musc_ between groups for any of the four common modules (Table 4). However, the W_sum_ of the QUAD module was greater in the KOA individuals than in the younger adults when all participants with the QUAD module were considered, regardless of modular control complexity (pairwise *p* = 0.025). When only individuals with 3 modules and a QUAD module were included in the analysis (excludes one younger adult with 4 modules), the individuals with KOA still had a greater W_sum_ than the younger adults (F_2,18_ = 3.97, *p* = 0.040; pairwise *p* = 0.037).

**Table 4:** W_musc_ and W_sum_ for common modules.

Module	Variable	Population			p-Value	Test Statistic
		Younger	Older	KOA		
QUAD	n^†^	9	7	4	-	
	W_musc_	7.7 ± 0.5	7.9 ± 0.4	7.5 ± 0.6	0.461	Χ^2^_(2, N = 20)_ = 1.55
	W_sum_	3.1 ± 0.5	3.5 ± 0.5	4.0 ± 0.6	0.026*	F_2,17_ = 4.56
HAMS	n^†^	10	6	3	-	
	W_musc_	7.9 ± 0.3	7.8 ± 0.4	7.7 ± 0.6	0.638	Χ^2^_(2, N = 19)_ = 0.90
	W_sum_	2.6 ± 0.4	2.3 ± 0.4	2.1 ± 0.7	0.231	F_2,16_ = 1.61
PF	n^†^	9	9	8	-	
	W_musc_	7.7 ± 0.5	7.6 ± 0.7	7.5 ± 0.5	0.796	Χ^2^_(2, N = 26)_ = 0.46
	W_sum_	3.2 ± 0.4	3.4 ± 0.4	3.3 ± 0.6	0.646	F_2,23_ = 0.45
QH	n^†^	0	3	4	-	
	W_musc_	-	8.0 ± 0.0	8.0 ± 0.0	1.000	Χ^2^_(1, N = 7)_ = 0.00
	W_sum_	-	4.8 ± 0.2	4.8 ± 0.5	0.855	t(5) = 0.19^a^

* Pairwise difference between Younger and KOA (*p* = 0.025).

^**†**^ n = number of participants within the population that have the common module.

^a^ A two-sample t-test was used to test for differences in W_sum_ for the QH module since it was only present in the older adults and individuals with KOA.

**Fig 6:**
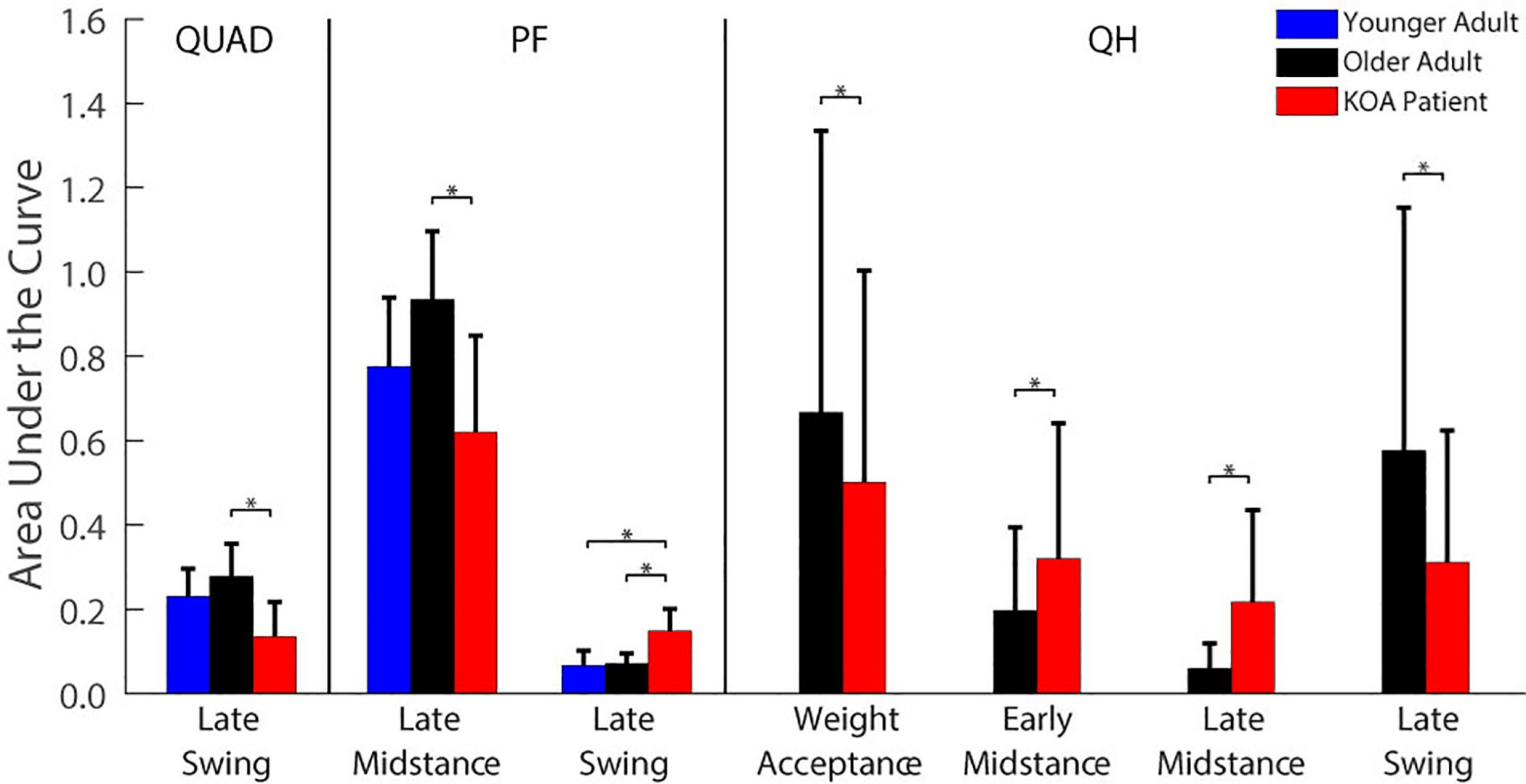
Area under the curve for common module activation timing profiles with significant differences between populations.

Module activation timing profiles differed between at least two groups for in each of the common modules, with the exception of the HAMS module (Fig 6). The older adults had greater AUC in the QUAD module during late swing than the KOA group (pairwise *p* = 0.018). In the PF module, the older adults had a greater late midstance AUC (pairwise *p* = 0.006), but a smaller late swing AUC than the KOA group (pairwise *p* = 0.001). The younger adults also had a smaller late swing AUC than the participants KOA (pairwise *p* < 0.001). In the QH module the older adults had a greater weight acceptance AUC (pairwise *p* = 0.043), but smaller early (pairwise *p* = 0.030) and late (pairwise *p* = 0.035) midstance AUCs than the individuals with KOA. There were no significant differences in AUC for all other gait phases in the common modules (all pairwise *p* ≥ 0.050).

### Joint kinematics and kinetics

**Fig 7:**
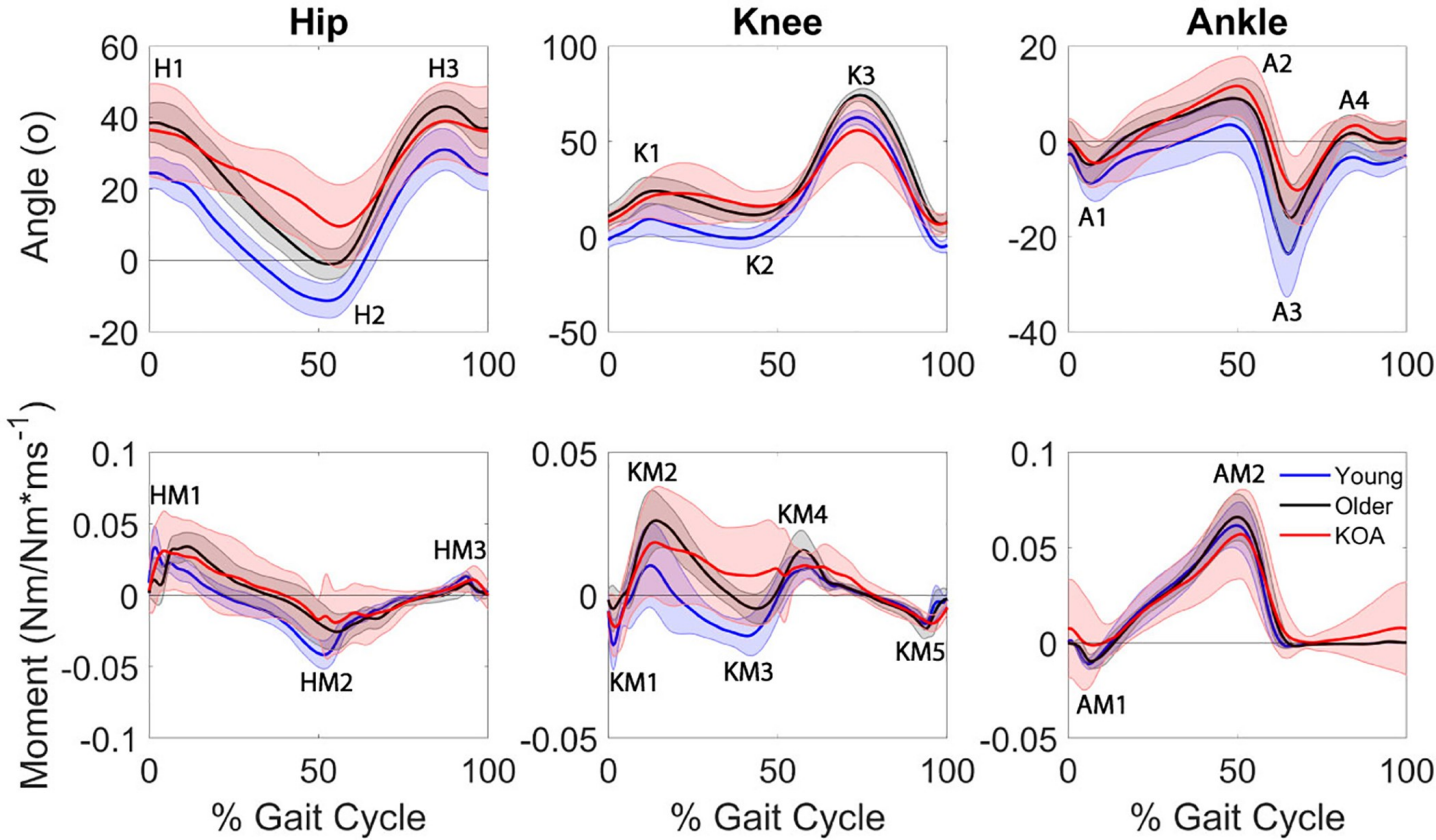
Population sagittal plane joint angles and joint moments (normalized to body weight and height). Solid lines represent population average and shaded error bars represent ± one standard deviation. Positive values represent hip flexion, knee flexion, and ankle dorsiflexion angles and hip extension, knee extension, and ankle plantarflexion moments.

There were several differences in peak joint kinematics and kinetics between populations (Table 5; Fig 7). Compared to the older adult and KOA groups, the younger adults had smaller peak hip flexion angles in early stance (H1: all pairwise *p* ≤ 0.001) and swing (H3: all pairwise *p* ≤ 0.018), greater hip extension (H2: all pairwise *p* ≤ 0.006), less knee flexion in early stance (K1: all pairwise *p* ≤ 0.015), greater knee extension (K2: all pairwise *p* ≤ 0.001), and less dorsiflexion during swing (A4: all pairwise *p* ≤ 0.005). The older adults had a greater peak knee flexion angle during late stance than the younger adults and individuals with KOA (K3: all pairwise *p* ≤ 0.027). The KOA group had a smaller peak hip extension angle than the older adults (H2: pairwise *p* = 0.014) and a greater peak late stance dorsiflexion angle (A2: pairwise *p* = 0.007) and a smaller peak plantarflexion angle (A3: pairwise *p* = 0.014) than the younger adults. With respect to joint kinetics, the younger adults had a greater hip flexion moment than the older adults and KOA participants (HM2: all pairwise *p* ≤ 0.018). The younger adults also had a greater midstance peak knee flexion moment (KM3: pairwise *p* = 0.001) than the individuals with KOA. Compared to the younger adults, the older adults had a smaller peak weight acceptance knee flexion moment (KM1: pairwise *p* = 0.018) and a greater early stance peak knee extension moment (KM2: pairwise *p* = 0.036). There were no differences between populations for all other peak joint angles and moments (all pairwise *p* ≥ 0.060).

**Table 5:** Peak joint angles and moments.

Metric	Joint	Peak	Younger (n = 10)	Older (n = 10)	KOA (n = 10)	p-Value	Test Statistic (F_2,27_)
Angle (^o^)	Hip	H1	24.7 ± 4.4	39.2 ± 5.7	39.5 ± 7.0	<0.001^a^^,^^b^	21.50
		H2	-11.5 ± 4.8	-1.3 ± 4.1	7.7 ± 9.6	<0.001^a^^,^^b^^,^^c^	20.83
		H3	31.4 ± 6.0	43.2 ± 4.5	40.2 ± 8.9	0.002^a^^,^^b^	8.40
	Knee	K1	9.6 ± 8.3	24.5 ± 7.2	24.4 ± 15.4	0.006^a^^,^^b^	6.16
		K2	-1.8 ± 5.5	11.2 ± 3.8	14.5 ± 8.0	<0.001^a^^,^^b^	20.42
		K3	62.8 ± 3.8	74.7 ± 3.6	57.7 ± 15.8	0.002^a^^,^^c^	8.21
	Ankle	A1	-9.4 ± 3.8	-5.4 ± 3.8	-6.8 ± 4.6	0.105	2.45
		A2	4.6 ± 5.3	9.7 ± 3.8	12.4 ± 6.3	0.008^b^	5.77
		A3	-25.2 ± 9.3	-17.7 ± 7.6	-13.9 ± 7.8	0.017^b^	4.79
		A4	-2.0 ± 2.7	2.8 ± 3.5	4.5 ± 2.9	<0.001^a^^,^^b^	11.98
Moment (x10^-2^ Nm/Nm*m/s^-1^)	Hip	HM1	3.7 ± 0.9	3.8 ± 0.9	3.8 ± 2.3	0.984	0.02
		HM2	-4.2 ± 0.9	-2.9 ± 0.7	-2.9 ± 1.0	0.004^a^^,^^b^	7.04
		HM3	1.4 ± 0.3	0.9 ± 0.5	1.4 ± 0.6	0.093	2.59
	Knee	KM1	-1.7 ± 0.8	-0.7 ± 0.7	-1.4 ± 0.9	0.023^a^	4.37
		KM2	1.1 ± 1.5	2.7 ± 1.0	2.3 ± 1.6	0.045^a^	3.48
		KM3	-1.6 ± 0.6	-0.6 ± 0.5	0.3 ± 1.5	0.002^b^	8.26
		KM4	1.1 ± 0.4	1.7 ± 0.5	1.4 ± 0.9	0.041	3.61
		KM5	-1.1 ± 0.2	-1.3 ± 0.4	-1.0 ± 0.3	0.086	2.69
	Ankle	AM1	-1.2 ± 0.3	-1.0 ± 0.4	-0.9 ± 0.4	0.357	1.07
		AM2	6.2 ± 1.0	6.6 ± 1.2	6.0 ± 2.2	0.620	0.49

Peak joint angles and moments reported in the table as the average ± standard deviation. Hip flexion, knee flexion, and ankle dorsiflexion *angles* are positive. Hip extension, knee extension, and ankle plantarflexion *moments* are positive and were normalized to body mass, height, and walking speed. Superscripted letters indicate pairwise differences between

^a^ Younger and Older.

^b^ Younger and KOA.

^c^ Older and KOA.

For those participants with fewer than 3 modules, merging of two typically independent common modules occurred. Of the 30 total participants, 20 had an independent QUAD module and 9 had a merged QUAD module, 19 participants had an independent HAMS module and 11 participants had a merged HAMS module, and 26 participants had an independent PF module and 3 participants had a merged PF module. One younger adult had two independent modules that described the activity of the quadriceps muscles and another younger adult had two independent modules that described the activity of the plantarflexor muscles. These two younger adults were excluded from the comparison of peak joint angles and moment by common module.

The merging of the HAMS module with another module or additional muscles affected peak joint angles and moments (Table 6). Individuals demonstrating a HAMS module that was merged with other muscles had greater hip flexion during early stance (H1; *p* = 0.039) and swing phase (H3; *p* = 0.038) and greater late stance dorsiflexion (A2; *p* = 0.048) than individuals not having a merged HAMS module. A merged HAMS module also resulted in a smaller knee flexion moment during midstance (K3; *p* = 0.030). A merged QUAD or PF module did not significantly alter peak joint angles or moments (*p* ≥ 0.057).

**Table 6:** Peak joint angles and moments by common module.

Metric	Joint	Peak	QUAD Module				HAMS Module				PF Module			
			Independent (n = 20)	Merged (n = 9)	p-value	Test Statistic t(27)	Independent (n = 19)	Merged (n = 11)	p-value	Test Statistic t(28)	Independent (n = 26)	Merged (n = 3)	p-value	Test Statistic t(27)
Angle (^o^)	Hip	H1	33.1 ± 9.1	39.5 ± 5.3	0.060	-1.97	31.9 ± 9.9	38.9 ± 5.0	0.039*	-2.16	34.1 ± 9.2	39.3 ± 8.5	0.363	-0.92
		H2	-3.4 ± 9.2	3.7 ± 10.6	0.074	-1.86	-4.4 ± 9.8	3.0 ± 9.7	0.053	-2.02	-2.6 ± 9.1	8.9 ± 16.5	0.066	-1.91
		H3	37.2 ± 9.0	42.1 ± 4.2	0.136	-1.54	35.9 ± 9.3	42.3 ± 3.9	0.038*	-2.18	37.8 ± 8.6	42.5 ± 5.8	0.366	-0.92
	Knee	K1	21.4 ± 14.3	17.5 ± 6.5	0.444	0.78	20.6 ± 15.4	17.6 ± 5.9	0.543	0.62	20.8 ± 12.5	17.3 ± 2.6	0.638	0.48
		K2	6.8 ± 10.2	11.5 ± 5.9	0.212	-1.28	6.2 ± 10.6	11.0 ± 5.5	0.169	-1.41	8.2 ± 9.0	12.3 ± 4.4	0.447	-0.77
		K3	66.0 ± 13.1	63.7 ± 9.2	0.629	0.49	65.3 ± 13.4	64.7 ± 8.8	0.897	0.13	65.6 ± 12.2	61.8 ± 11.3	0.559	0.59
	Ankle	A1	-7.6 ± 4.2	-6.1 ± 4.7	0.376	-0.90	-7.8 ± 4.3	-6.2 ± 4.3	0.327	-1.00	-6.7 ± 3.7	-7.5 ± 4.7	0.724	0.36
		A2	7.6 ± 6.4	12.2 ± 3.9	0.057	-1.99	7.2 ± 6.3	11.7 ± 4.3	0.048*	-2.07	8.8 ± 6.3	10.2 ± 5.5	0.723	-0.36
		A3	-20.9 ± 9.2	-15.7 ± 8.9	0.168	-1.42	-19.8 ± 9.6	-17.5 ± 8.9	0.518	-0.65	-19.0 ± 8.9	-13.8 ± 11.1	0.359	-0.93
		A4	1.2 ± 4.5	3.5 ± 2.8	0.158	-1.45	0.9 ± 4.4	3.2 ± 3.0	0.134	-1.54	2.0 ± 4.0	2.0 ± 3.2	0.999	<0.01
Moment (x10^-2^ Nm/Nm*ms^-1^)	Hip	HM1	3.7 ± 1.5	3.9 ± 1.6	0.778	-0.28	3.8 ± 1.5	3.7 ± 1.5	0.923	0.10	3.7 ± 1.3	4.2 ± 3.0	0.637	-0.48
		HM2	-3.3 ± 1.1	-3.2 ± 0.8	0.703	-0.39	-3.4 ± 1.3	-3.3 ± 0.7	0.701	-0.39	-3.3 ± 1.1	-4.2 ± 0.4	0.173	1.40
		HM3	1.2 ± 0.6	1.3 ± 0.5	0.724	-0.36	1.1 ± 0.5	1.4 ± 0.6	0.109	-1.66	1.2 ± 0.6	1.4 ± 0.5	0.590	-0.55
	Knee	KM1	-1.3 ± 0.9	-1.3 ± 0.9	0.810	0.24	-1.3 ± 0.9	-1.3 ± 0.9	0.889	-0.14	-1.2 ± 0.8	-1.6 ± 1.7	0.500	0.68
		KM2	2.2 ± 1.4	1.9 ± 1.5	0.601	0.53	2.0 ± 1.6	2.1 ± 1.4	0.859	-0.18	2.1 ± 1.4	2.4 ± 1.7	0.748	-0.32
		KM3	-0.9 ± 1.0	0.0 ± 1.5	0.061	-1.96	-1.0 ± 1.0	0.0 ± 1.3	0.030*	-2.29	-0.7 ± 1.2	0.5 ± 0.7	0.101	-1.70
		KM4	1.5 ± 0.7	1.6 ± 0.7	0.578	-0.56	1.5 ± 0.7	1.5 ± 0.7	0.832	-0.21	1.5 ± 0.6	2.0 ± 0.9	0.162	-1.44
		KM5	-1.2 ± 0.4	-1.1 ± 0.2	0.665	-0.44	-1.1 ± 0.4	-1.2 ± 0.2	0.647	0.46	-1.1 ± 0.3	-1.3 ± 0.2	0.375	0.90
	Ankle	AM1	-1.1 ± 0.4	-0.9 ± 0.3	0.215	-1.27	-1.1 ± 0.4	-0.9 ± 0.3	0.146	-1.49	-1.0 ± 0.4	-0.9 ± 0.3	0.494	-0.69
		AM2	6.1 ± 1.6	6.5 ± 1.4	0.575	-0.57	6.2 ± 1.7	6.4 ± 1.3	0.700	-0.39	6.2 ± 1.5	7.4 ± 1.8	0.218	1.26

Peak joint angles and moments reported as the average ± standard deviation. Hip flexion, knee flexion, and ankle dorsiflexion *angles* are positive. Hip extension, knee extension, and ankle plantarflexion *moments* are positive and were normalized to body mass, height, and walking speed.

* indicates statistical differences due to the absence of a common module.

### Associations between module number and clinical and performance metrics

Self-selected walking speed was positively associated with the number of required modules when all participants were grouped together such that individuals who walked faster required a greater number of modules (*τ*_*b*_ = 0.350, *p* = 0.021; Table 7). However, within a population, only the KOA group had a significant positive correlation between the number of required modules and self-selected walking speed (*τ*_*b*_ = 0.581, *p* = 0.045). There were no signficant associations between the number of required modules and self-selected walking speed for the younger or older adults (*p* ≥ 0.649).

**Table 7:** Self-selected walking speed* (m/s) by population and number of modules.

Population	Number of Modules				By Population		All Participants	
	1	2	3	4	*τ*_*b*_	*p*-value	*τ*_*b*_	*p*-value
Younger			1.23 ± 0.14	1.30 ± 0.28	0.113	0.793	0.350	0.021^a^
Older		1.18 ± 0.16	1.11 ± 0.22		-0.163	0.649		
KOA	0.56 ± 0.00	0.93 ± 0.17	1.08 ± 0.11		0.581	0.045^a^		

Abbreviations: *τ*_*b*_, Kendall rank correlation coefficient.

* Speed reported as average ± standard deviation.

^a^ significant correlation.

There was a significant association between the number of modules required by the KOA group and the ADL subscale of the KOOS (*τ*_*b*_ = 0.644, *p* = 0.021) with higher (better) scores associated with a greater number of modules (Table 8). There were no significant correlations between the number of required modules and other KOOS subscales (all *p* ≥ 0.055), KL grade (*p* = 0.316), or the performance-based clinical assessments (all *p* ≥ 0.133).

**Table 8:** Knee osteoarthritis clinical assessments by module group.

Number of Modules	KL Grade	Performance-Based Outcomes			KOOS Scores			
		6MW (m)	SCT (s)	TUG (s)	Pain	Symptoms	ADL*	QOL
One Modules (n = 1)	4.0 ± 0.0	180.4 ± 0.0	39.9 ± 0.0	15.4 ± 0.0	39.0 ± 0.0	50.0 ± 0.0	40.0 ± 0.0	25.0 ± 0.0
Two Modules (n = 5)	3.0 ± 0.0	379.4 ± 84.8	33.7 ± 17.4	10.9 ± 2.9	42.0 ± 12.1	44.4 ± 17.8	53.2 ± 11.0	22.8 ± 18.1
Three Modules (n = 4)	3.8 ± 0.5	434.0 ± 80.5	18.3 ± 14.7	9.4 ± 0.4	74.3 ± 21.3	67.8 ± 20.7	75.5 ± 18.3	47.0 ± 28.5

Abbreviations: KL, Kellgren Lawrence; 6MW, six-minute walk test; SCT, stair climbing test; TUG, timed-up and go test; KOOS, knee injury and osteoarthritis outcome score; ADL, activities of daily living; QOL, quality of life.

* Significant positive association with number of modules (*p* = 0.021).

## Discussion

As a step toward distinguishing between changes to the neuromuscular control of gait due to healthy aging and changes due to age-related pathologies, this pilot study aimed to determine whether age and KOA alter the modular control of walking. The experimental activation patterns of all participants were able to be reduced to a modular organization. Thus, the findings of this study contribute to the evidence for a low-dimensional organization of the neural control of muscles during gait across age groups and in the presence of pathology. Individuals with KOA required fewer modules to adequately reconstruct their experimental activation patterns than younger adults. However, the number of modules required by the older adults did not significantly differ from the other two populations. In addition, the VAF, which indicates the amount of variability in the muscle activation patterns, was greater in individuals with KOA than the younger and older adults when one and two modules were extracted and greater than the younger adults when 3–7 modules were extracted. In contrast, the older adults marginally (on average, 0.4–1.4%) greater VAF than the younger adults when 4–6 modules were extracted suggests a slight decrease in muscle activation variability due to age. Together, the number of modules and VAF suggest that aging alone may not produce a significant reduction in modular control complexity. However, the combined effects of KOA and advanced age on neuromuscular control may together reduce the modular control complexity of walking.

**Fig 8:**
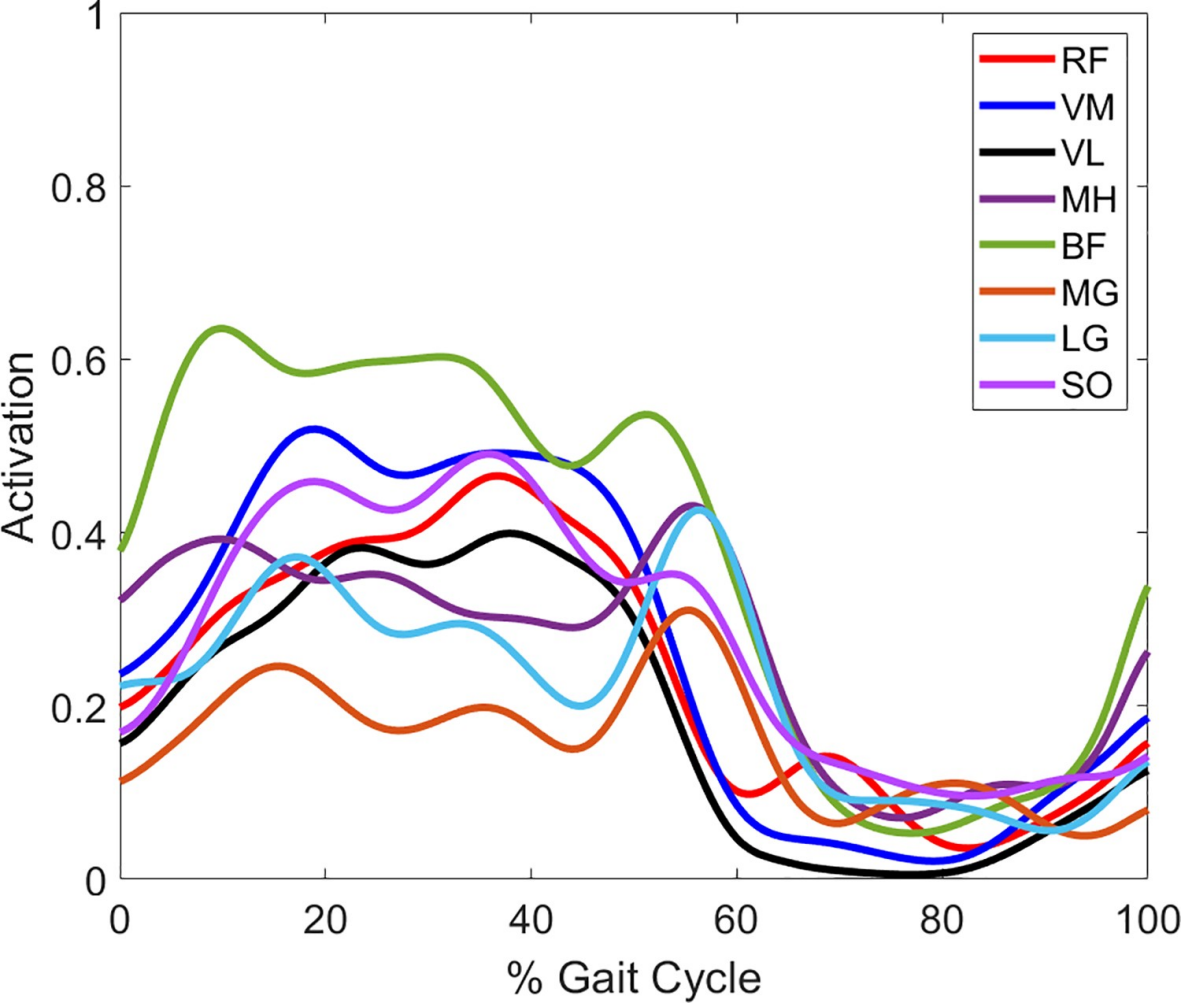
Average muscle EMG for the KOA participant with one module (KOA 10). Muscle abbreviations: rectus femoris (RF), vastus medialis (VM), vastus lateralis (VL), medial hamstrings (MH), biceps femoris (BF), medial gastrocnemius (MG), lateral gastrocnemius (LG), soleus (SO).

The number of required modules in each population in this study (younger: 3–4, older: 2–3, KOA: 1–3) was generally consistent with previous analyses of modular control during walking in healthy younger and older adults and patient populations (e.g., [24, 31, 46, 47]). The selection of muscles included in a module analysis influences the resulting modules [47]. Thus, with the muscle set collected in this study comprised of three major muscle groups (the quadriceps, hamstrings, and plantarflexors), it is reasonable that three modules are sufficient for independent control of these muscles. Indeed, 19 of the 30 total participants required three modules. Only two younger adults required more than three modules, which may provide greater flexibility in an individual’s control strategy. For example, independent control of the biarticular rectus femoris and uniarticular vasti (Fig 3: young adult (YA) 09) would promote greater independence of the hip flexor and knee extensor actions. Most participants with two modules exhibited merged quadriceps and hamstrings activation, with the exception of one KOA participant who exhibited merged activation of all muscles except for the biceps femoris (Fig 5: KOA 09). One KOA patient only required one module to adequately reconstruct the activation patterns of all eight muscles (Fig 5: KOA 10). This individual exhibited a relatively constant activation of all eight EMG muscles throughout the stance phase (Fig 8), which is indicative of a very simplistic “all or nothing” control strategy that activates all or none of the muscles at a given time.

The three common modules identified in the younger adults (i.e., QUAD, HAMS, and PF) were also observed in a majority of the older adults. For those younger and older adults with the common modules, the module compositions were very strongly correlated between age groups (*r* ≥ 0.970). There were no differences in muscle activation timing between the younger and older adults’ modules. In addition, W_sum_ did not differ between the healthy younger and older adults for any of the common modules, which indicates that, when those independent modules are present, muscle co-activity does not increase significantly. Together, these findings suggest the nervous system may be capable of preserving modular control with unimpaired aging.

It is important to note that there was no statistically significant difference in walking speed between the younger and older adults in this study, which suggests the older adults in this study had a relatively high level of function. Although consistent modular organization has been observed across walking speeds in healthy younger and older adults [23, 24], the number of required modules was significantly associated with self-selected walking speed when all participants in this study were considered (Table 7). In addition, previous studies have reported an association between slower walking speeds and altered modular organization in patient populations [24, 45]. Therefore, future studies should investigate modular control in older adults with slower self-selected walking speeds to clarify the relationship between age-related changes in motor control and functional performance.

The KOA group exhibited altered module composition and timing compared to the unimpaired younger and older adults, which suggests these differences in modular control may be disease-related. Only four of the 10 participants with KOA had independent QUAD and HAMS modules (compared to seven of the 10 older adults). The other six participants with KOA exhibited merging of the quadriceps muscles with the hamstrings and/or plantarflexors. Those individuals with KOA with an independent QUAD module had a greater W_sum_ than the younger adults, which suggests that even when the QUAD module is independent in the KOA group (i.e., not merged with the HAMS module or other muscles), there is still an increase in co-activity of other muscles with the quadriceps muscles. These findings are consistent with the increased co-contraction of the quadriceps and hamstrings and of the quadriceps and gastrocnemius observed in individuals with KOA, especially in the more severe stages of the disease [48]. Compared to the three older adults with a QH module, the four KOA participants with a QH module had greater module activation during early and late midstance, the phases of the gait cycle when the affected leg is primarily responsible for supporting the body [49]. This is in contrast to the smaller QH module activation observed in the KOA group during late swing and weight acceptance when they would have been fully or partially supported by the contralateral limb. The increased QH activation during midstance observed in the KOA group but not the unimpaired older adults may be a disease-related control strategy to stabilize the knee during single-leg support. Thus, although the QH module composition was very strongly correlated between the older adults and KOA group (*r* = 0.990), the module activation pattern was significantly influenced by the presence of KOA.

Notably, the number of significantly active muscles per module (W_musc_) did not differ between populations for any of the common modules. The average W_musc_ was greater than or equal to 7.5 for all populations and all common modules, indicating that the weight of most or all muscles was significantly different from 0 and, therefore, statistically associated with the module. However, visual inspection of the muscle weights for the common modules for each population in Figs 3–5 shows that the contributions outside of the primary muscles associated with the module were generally minimal. A clear exception is the QUAD module of the KOA group, for which multiple muscles in addition to the quadriceps had large weights, which is consistent with their larger W_sum_.

The absence of the independent HAMS module resulted in greater peak hip flexion and ankle dorsiflexion angles and a smaller peak midstance knee flexion moment. The KOA groups’ prolonged knee extension moment during midstance compared to the flexion moment observed in the younger adults is likely related to the high prevalence of merging of the control of the hamstring muscles with the quadriceps and plantarflexors in the individuals with KOA. The increased midstance activation of the QH module (when the HAMS module is merged with the QUAD module), along with the larger W_sum_ when the QUAD module was present in the participants with KOA, likely contribute to the KOA groups’ midstance knee extension moment by increasing the contribution of the quadriceps to knee extension that would otherwise decrease during midstance [49]. Thus, merging of typically independent modules can alter joint moment production ability, likely due to the inappropriately timed activation of antagonist muscles.

The altered neuromuscular control strategy identified in the KOA group in this study suggests that the reduced modular control complexity observed in individuals who had undergone a TKA [31] may have been present prior to surgery. Thus, replacement of the joint structure alone may not be sufficient to improve locomotor performance in individuals with end-stage KOA. However, additional research is required to determine if pre-operative modular control is associated with post-operative modular control. Retraining the neuromuscular control strategy may be required to achieve improvements in walking speed and coordination in individuals with KOA or following a TKA. Gait retraining has been shown to increase the number of modules and improve the quality of the modular organization and timing in individuals with post-stroke hemiparesis [50] and reduce co-contraction and knee pain in individuals with KOA [51]. Therefore, rehabilitation protocols aimed at appropriately timed muscle activity may be necessary to restore independent activation of these muscle groups in individuals following a TKA.

In contrast to previous studies which have reported associations between modular organization and functional performance in impaired populations [24, 31], the number of modules required to reconstruct the EMG of the participants with KOA was not associated with the clinical assessment measures, with the exception of a moderate correlation with their self-reported pain on the KOOS. Only EMG from the involved limb of the KOA group was included in this pilot study. While symmetry is a reasonable assumption in healthy adults, KOA commonly affects one knee more than the other. There may be differences in modular control between the involved limb and the contralateral limb which could provide insight into the relationship between clinical scores and motor control complexity. In addition, a limitation of this pilot study is the small sample size. In this secondary analysis of previously collected data, our sample size of 10 participants per group provided a statistical power of 0.72 to detect differences in module number between groups. A power analysis indicated that a sample size of 12 participants per group would have provided a statistical power of 0.80; however, our cohorts were not that large for each of our three study groups. Still, we detected a significant difference in module number between the younger adults and individuals with KOA, suggesting these findings were not subject to a type II error. In addition, though not statistically significant, there were trends of better average performance-based outcomes and KOOS symptom, ADL, and QOL subscales with a greater number of modules (Table 8). Based on the findings of this study, a post-hoc power analysis indicated that a sample size of 8 individuals with KOA per module group would be required to detect differences in ADL KOOS scores between module groups with a power of 0.80. Thus, the trends observed in this study suggest that a larger study with a sufficient sample size in each module group is warranted to determine if modular control complexity is associated with measured or self-reported function in individuals with KOA. Still, the findings of this study suggest there are changes to the neuromuscular control of walking that occur in the presence of severe KOA which may not be corrected by the structural replacement of joint components in a TKA.

In conclusion, this study is the first to reveal alterations in the modular control of walking in the presence of KOA. In contrast, modular control complexity and organization was similar between healthy age groups, which suggests that healthy aging alone does not significantly alter the neuromuscular control strategy of walking. The participants with KOA exhibited decreased module complexity and altered module composition characterized by increased muscle co-activity, particularly of the quadriceps and hamstrings. This loss of modular control complexity may contribute to slower walking speeds, altered joint kinematics and kinetics, and increased self-reported pain. Importantly, the participants with KOA in this study were scheduled for a TKA. While a TKA replaces the structural components of the joint, the effects of the surgery itself or the rehabilitation following surgery on neuromuscular control remain unclear. Therefore, the findings of this pilot study, which suggest a change in underlying neuromuscular control strategy associated with the presence of KOA, may have important implications for the inclusion of gait retraining targeting appropriately timed neuromuscular activity in TKA rehabilitation protocols. Future work is needed to clarify the potential relationship between KOA-related changes in modular control strategy and functional outcomes.
